# miR-142-3p in cancer: intracellular tumor suppression and extracellular vesicle- mediated microenvironmental signaling

**DOI:** 10.1007/s11033-026-12631-y

**Published:** 2026-08-29

**Authors:** Michelle van der Merwe, Rebecca Towle, Kathy Myburgh, Cathie Garnis, Anna-Mart Engelbrecht

**Affiliations:** 1 Department of Physiological Sciences, Stellenbosch University, Stellenbosch, South Africa; 2 Department of Basic and Translational Research, British Columbia Cancer Research Centre, Vancouver, BC Canada

**Keywords:** miR-142-3p, Cancer, Treatment resistance, Extracellular vesicles, miRNA, Treatment resistance, Tumor microenvironment

## Abstract

miR-142-3p has emerged as an important regulator of cancer progression and treatment response, exhibiting predominantly tumor-suppressive functions. Intracellularly, miR-142-3p inhibits proliferation, promotes apoptosis, suppresses epithelial–mesenchymal transition (EMT), and enhances chemosensitivity through the downregulation of key pathways, including HMGB1, Wnt/β-catenin, and SIRT1-mediated autophagy. Notably, miR-142-3p is frequently enriched in extracellular vesicles (EVs), indicating selective export from tumor cells. This EV-mediated trafficking introduces functional complexity, as the loss of intracellular miR-142-3p may attenuate its tumor-suppressive effects, while its transfer to recipient cells within the tumor microenvironment (TME) may exert context-dependent roles. Within the TME, miR-142-3p has been associated with the regulation of cancer-associated fibroblast (CAF) activation, angiogenesis, and immune responses. Emerging evidence further suggests a potential role for EV-associated miR-142-3p in systemic processes such as cancer-associated cachexia through modulation of muscle-related pathways, although this remains poorly defined. This review summarizes the dual intracellular and extracellular functions of miR-142-3p in cancer and highlights its contribution to treatment sensitivity and TME interactions. We also discuss current challenges and future directions, including therapeutic strategies aimed at restoring intracellular miR-142-3p levels or modulating its EV-mediated transfer. A deeper understanding of its context-dependent roles will be critical for the development of targeted and clinically relevant therapeutic approaches.

**Graphical Abstract:**

**Figure:**
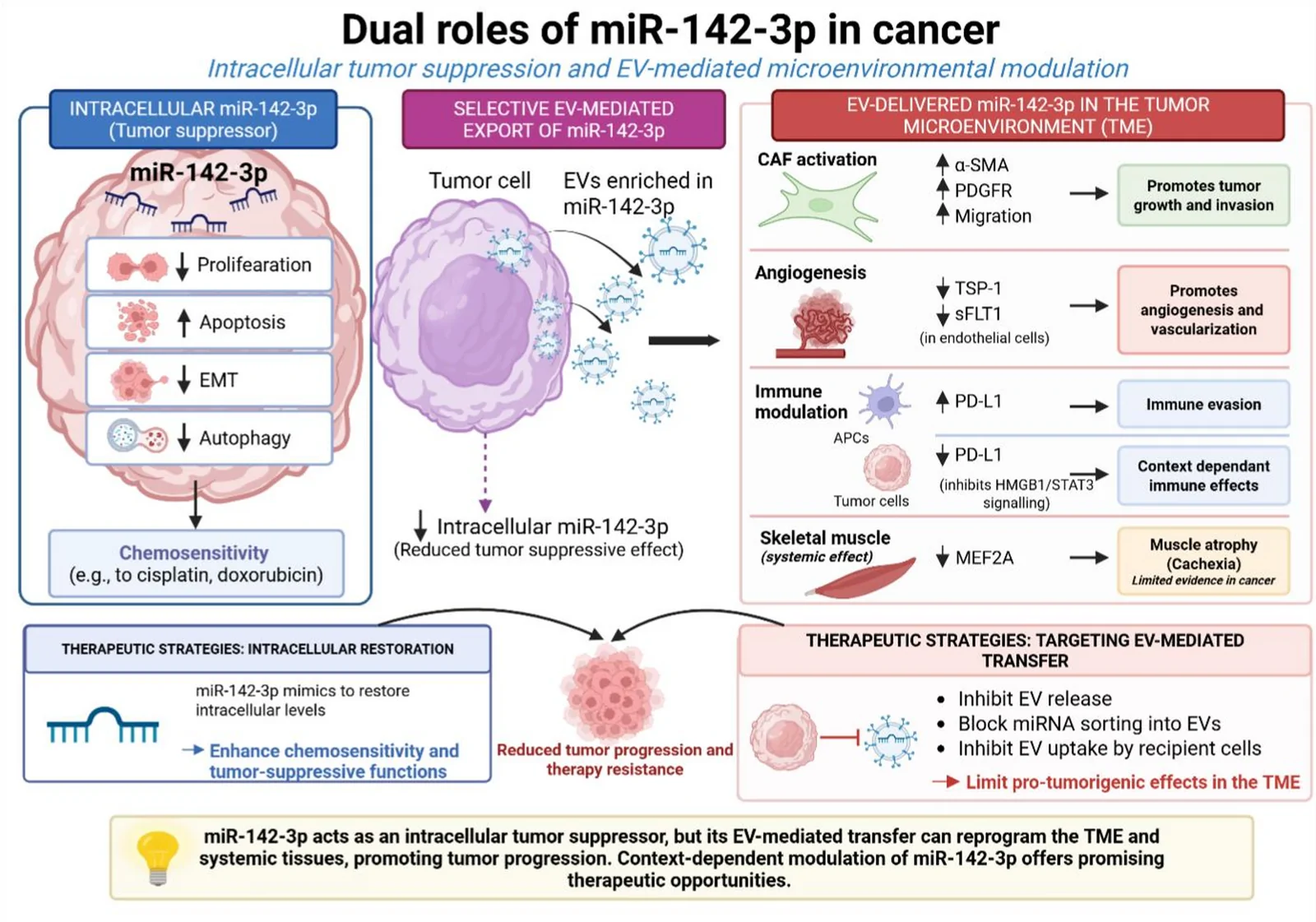

## Introduction

Cancer continues to represent a major global health burden, with its incidence projected to increase by approximately 77%, from 20 million cases in 2022 to nearly 35 million cases by 2050 [1]. Despite advances in treatment, tumor progression and treatment resistance remain major barriers to therapeutic success. Both processes are driven by interconnected mechanisms, including enhanced survival signaling, epithelial-to-mesenchymal transition (EMT), increased drug efflux, autophagy, and interactions within the tumor microenvironment (TME) [2]. MicroRNAs (miRNAs), small non-coding RNAs typically 18–22 nucleotides in length, are important post-transcriptional regulators of these mechanisms and can influence cancer progression and treatment response through multiple target genes and signaling pathways [3–8].

miRNA biogenesis begins in the nucleus, where RNA polymerase II transcribes miRNA genes into primary miRNAs. These transcripts are processed by the Drosha–DGCR8 Microprocessor complex, exported to the cytoplasm by exportin-5 (XPO5), and further cleaved by Dicer to generate miRNA duplexes [9]. One strand is preferentially loaded onto an Argonaute (AGO) protein to form the RNA-induced silencing complex (RISC), whereas the passenger strand is typically degraded. The canonical mechanism of miRNA action involves recognition of complementary miRNA response elements (MREs) within the 3′ untranslated regions (UTR) of target mRNAs, generally resulting in mRNA destabilization or translational repression [9]. However, functional MREs may also occur within 5′-UTR and coding sequences, representing non-canonical modes of miRNA regulation [10, 11]. Mature miRNAs and AGO-containing complexes have also been detected in the nucleus, where they may interact with RNA transcripts or genomic loci and influence transcription-factor recruitment, DNA methylation, histone modifications, and chromatin accessibility [11–14]. Depending on the target and cellular context, nuclear miRNAs may promote either transcriptional silencing or activation [15].

miRNA expression is frequently dysregulated in cancer due to genomic alterations and disrupted transcriptional regulation, while intracellular levels may also be altered by selective packaging and export through extracellular vesicles (EVs) [16–19]. Tumor-derived EV miRNAs have been shown to regulate immune-cell activity, cancer-associated fibroblast activation, and angiogenesis [20–27]. Importantly, the biological effect of a miRNA can depend on the molecular context of the host cell. For example, miR-126 can suppress cancer-cell proliferation while promoting endothelial-cell proliferation, migration, and angiogenesis through cell-specific differential regulation of the PI3K/AKT pathway [28, 29].

miR-142-3p is relevant to this context-dependent regulation. Within cancer cells, miR-142-3p generally functions as a tumor suppressor by regulating proliferation and survival, EMT, drug efflux, and autophagy [3, 30–37]. However, following EV mediated transfer to cells within the TME, miR-142-3p can exert distinct and sometimes tumor-promoting effects, including the promotion of angiogenesis and cancer-associated fibroblast activation [4, 38, 39]. Furthermore, miR-142-3p has been implicated in immune regulation and myogenic pathways relevant to cachexia, although evidence for these effects in cancer-specific EV models remains limited [40–43]. The function of miR-142-3p may therefore depend on its cellular abundance, localization, and EV-mediated transfer. Several studies have reported the selective packaging and export of miR-142-3p through EVs, resulting in preferential EV enrichment in some cancer models [38, 39, 44].

Two previous reviews provide important foundations for the present work. Zareifar et al. (2024) reviewed the functions of both miR-142-3p and miR-142-5p across cancer types, including their roles in signaling and immunomodulation [45]. Our previous review examined the selective EV export of several tumor-suppressive miRNAs specifically in cervical cancer [46]. Building on this work, the present review focuses exclusively on miR-142-3p across cancer types, bringing together evidence on its intracellular functions and the consequences of its selective EV export and transfer to cells within the TME.

## Intracellular functions of miR-142-3p in cancer

Consistent with its predominantly tumor-suppressive intracellular role, miR-142-3p expression is reduced across several cancer types. In lung adenocarcinoma, miR-142-3p was found to be downregulated in approximately 52% of tumors compared to adjacent normal tissue and was also reduced in lung cancer cell lines [47–49]. Lower expression was associated with larger tumors, advanced stage, lymph-node metastasis, and treatment resistance [49]. Reduced miR-142-3p expression has similarly been reported in breast cancer tissue and cell lines [37, 50], colorectal cancer tissues [51], and gastric and ovarian cancers [3, 31]. These expression patterns provide a basis for examining the intracellular mechanisms through which miR-142-3p restrains tumor progression.

### Regulation of proliferation and apoptosis

The inhibitory effects of miR-142-3p on proliferation and its pro-apoptotic activity have been reported across multiple cancer types, supporting its predominant tumor-suppressive role within cancer cells.

One recurrent mechanism through which miR-142-3p inhibits proliferation is the suppression of high mobility group box 1 (HMGB1). HMGB1 is known to promote tumor progression by activating multiple oncogenic pathways, including NF-κB, MAPK/ERK, and PI3K/Akt signaling through interactions with RAGE and Toll-like receptors [52]. In cervical cancer, miR-142-3p has been shown to suppress cell proliferation and promote apoptosis via direct targeting of HMGB1, as well as additional members of the high-mobility group family, including HMGA1, HMGA2, and HMGB3 [53, 54]. Direct binding of miR-142-3p to the 3′-UTR of HMGB1 mRNA was demonstrated, supporting HMGB1 as a functional target. HMGB1 downregulation was accompanied by activation of pro-apoptotic markers, including cleaved PARP, caspase-3, caspase-7, caspase-9, and BAX, alongside reduced expression of anti-apoptotic proteins such as Bcl-2 and Bcl-xL in miR-142-3p-overexpressing cervical cancer cells [54]. Similar tumor suppressive effects have been observed in other malignancies. In glioma, miR-142-3p reduces cell viability and induces apoptosis through suppression of HMGB1 [33]. EV-mediated delivery of miR-142-3p to glioblastoma cells similarly suppressed HMGB1 after uptake, resulting in reduced proliferation and increased apoptosis [55].

Additionally, miR-142-3p exerts tumor-suppressive effects through HMGB1-independent mechanisms. In leukemia, miR-142-3p promoted apoptosis by targeting CIAPIN1, an anti-apoptotic protein involved in STAT3-mediated survival signaling and redox homeostasis, thereby reducing cell survival and increasing sensitivity to apoptotic stimuli [56, 57]. In colorectal cancer, miR-142-3p induced apoptosis partly through inhibition of the Rac1/ERK signaling pathway [51], while in lung cancer cell lines, it enhanced caspase-3 activation and promoted apoptotic cell death [48]. In cervical cancer, miR-142-3p overexpression significantly reduced cell proliferation and promoted apoptosis by downregulating frizzled 7 (FZD7) at both mRNA and protein levels [32]. Consequently, the oncogenic Wnt/β-catenin signaling pathway was inhibited. Furthermore, in breast cancer cells, miR-142-3p overexpression has been associated with reduced viability and increased apoptosis [37]. Collectively, these studies identify several intracellular pathways through which miR-142-3p can restrict tumor-cell growth and facilitate apoptosis, with HMGB1, CIAPIN1, RAC1/ERK, and FZD7/Wnt/β-catenin representing important context-dependent targets.

### Inhibition of epithelial–mesenchymal transition (EMT) and metastasis

EMT contributes to treatment resistance, tumor aggressiveness, and poor clinical outcomes across multiple cancer types [58–61]. During this process, cancer cells lose epithelial characteristics, including cell–cell adhesion and apical–basal polarity, while acquiring mesenchymal features that promote motility, invasion, and metastatic dissemination [62, 63].

miR-142-3p has generally been associated with suppression of EMT, migration, and invasion across cancer types. In cervical cancer, overexpression of miR-142-3p consistently increased E-cadherin while it reduced mesenchymal markers such as vimentin, N-cadherin, Snail, and ZEB1, resulting in decreased migration and invasion in vitro and reduced metastatic dissemination in vivo [32, 54]. Mechanistically, these effects were largely attributed to inhibition of Wnt/β-catenin signaling through the targeting of FZD7. This prevented β-catenin nuclear translocation and downstream transcription of EMT-associated transcription factors [32, 54]. In addition, miR-142-3p has been shown to suppress NF-κB activity, which may further contribute to EMT inhibition given the established role of NF-κB in driving the transcription of Snail, Slug, and TWIST [54].

In breast cancer, miR-142-3p suppressed migration via inhibition of the Wnt/β-catenin pathway through targeting of CXCL12, leading to decreased Snail and increased E-cadherin expression [37]. Furthermore, in another breast cancer study, miR-142-3p suppressed migration through targeting BACH1 thereby inhibiting EMT proteins such as MMP9 and CXCR4 [50]. In colorectal cancer, miR-142-3p overexpression reduced migration by inhibiting the RAC1/PERK pathway. This resulted in decreased expression of mesenchymal markers, including vimentin, N-cadherin, and MMP9, as well as increased E-cadherin [51]. Similarly, in lung cancer, miR-142-3p overexpression reduced migration, while its inhibition had the opposite effect [48]. Mechanistically, miR-142-3p was shown to directly target NR2F6 via 3′-UTR binding, consistent with the inverse correlation between miR-142-3p and NR2F6 expression observed in lung adenocarcinoma tissue. Additionally, MMP2 and MMP9 were also downregulated by miR-142-3p [48]. Notably, the pro-migratory effect induced by miR-142-3p knockdown was abolished by treatment with the MMP inhibitor BB-94, confirming that miR-142-3p regulates migration, at least in part, through modulation of MMP activity [48].

### Regulation of treatment sensitivity

Multiple studies indicate that miR-142-3p can enhance chemosensitivity across several malignancies, including breast, lung, ovarian, and non-small cell lung cancer (Table 1) [31, 36, 49, 64, 65]. Lower intracellular miR-142-3p expression, as well as higher levels in EV derived from chemoresistant cells, have been reported relative to chemosensitive counterparts [35, 49, 66].

Among these mechanisms, inhibition of treatment-induced protective autophagy has been identified as one mechanism through which miR-142-3p may enhance chemosensitivity. Although autophagy has context-dependent functions in cancer, it can enable tumor cells to withstand therapeutic stress by removing damaged cellular components and maintaining cellular homeostasis [64]. HMGB1 can promote autophagy through regulation of mammalian target of rapamycin (mTOR) signaling and Beclin-1-dependent autophagosome formation [67, 68]. Notably, chemotherapy-induced HMGB1 activation can promote protective autophagy and thereby contribute to treatment resistance [64, 69].

In non-small-cell lung cancer, miR-142-3p enhanced sensitivity to cisplatin and doxorubicin by targeting the 3′-UTR of HMGB1, activating mTOR, and suppressing protective autophagy [64]. These effects were also demonstrated in vivo, where tumors overexpressing miR-142-3p exhibited reduced HMGB1 expression and greater responsiveness to chemotherapy [64]. Similarly, miR-142-3p increased doxorubicin sensitivity in breast cancer cells through suppression of the HMGB1–autophagy axis [35].

SIRT1 represents another target linking miR-142-3p to treatment response. In ovarian cancer, miR-142-3p sensitized cisplatin-resistant SKOV3 cells by targeting SIRT1 [31]. As SIRT1 regulates autophagy-related proteins, including ATG7 and LC3, altered autophagy may contribute to this effect, although this mechanism was not directly established in this model [70]. Additionally, in H1299 lung cancer cells, miR-142-3p-mediated suppression of SIRT1 was directly associated with reduced autophagy and increased cisplatin sensitivity, while restoration of SIRT1 reversed these effects [49].

**Fig. 1:**
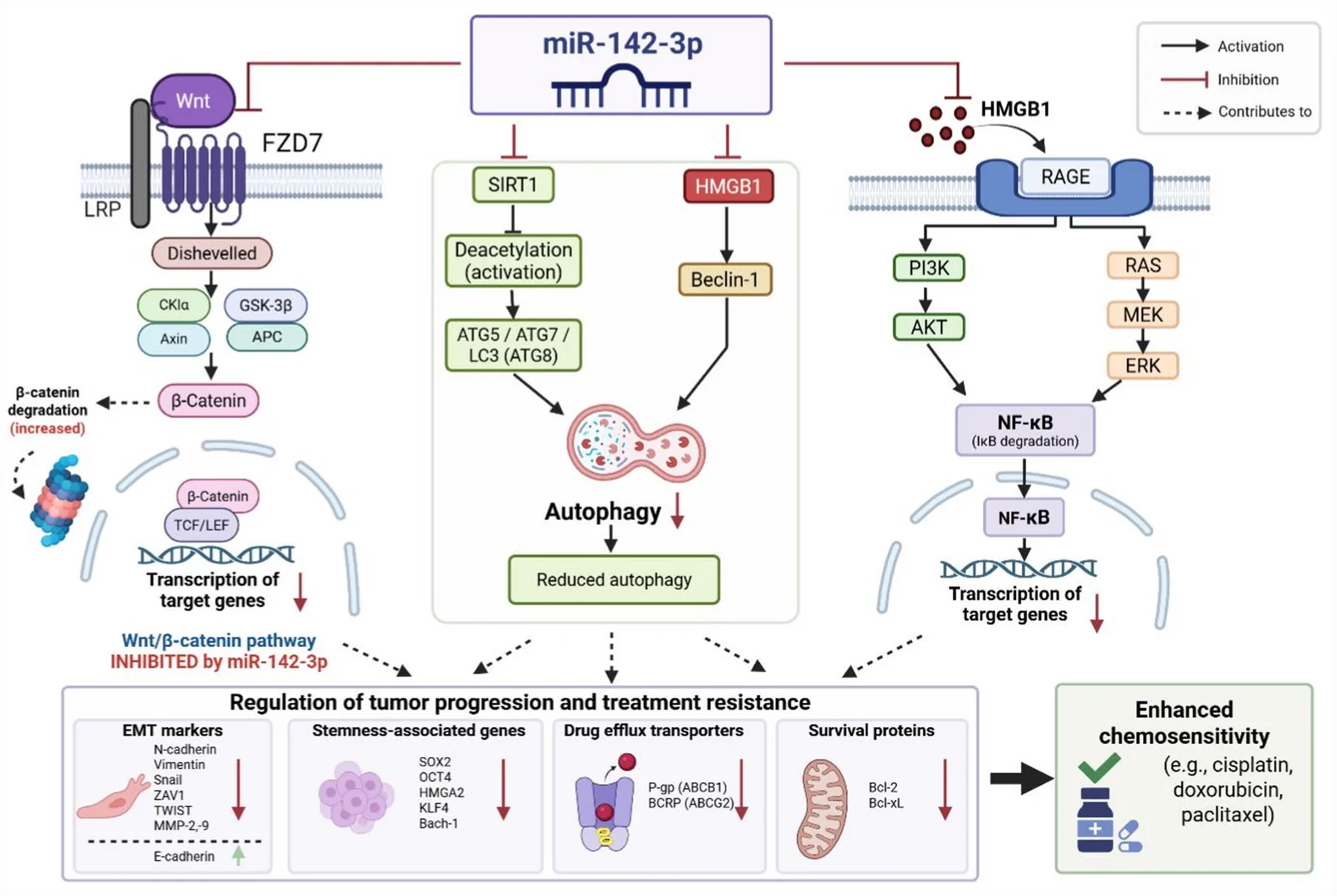
Mechanistic overview of miR-142-3p in cancer suppression and chemotherapy sensitization. miR-142-3p suppresses tumor progression and enhances chemotherapy response through multiple pathways. It inhibits Wnt/β-catenin signaling by downregulating FZD7, reducing downstream oncogenic transcription. It also targets HMGB1, thereby disrupting HMGB1–RAGE signaling and inhibiting PI3K/AKT and MAPK/ERK survival pathways, as well as Beclin-1–mediated autophagy. In addition, miR-142-3p regulates autophagy through inhibition of SIRT1. *Figure created with Biorender.com*

Beyond autophagy, miR-142-3p can influence chemosensitivity through the regulation of apoptosis, EMT, stemness, and drug efflux. In breast cancer, combining miR-142-3p with docetaxel reduced migration and enhanced apoptosis, accompanied by decreased expression of oncogenic and stemness-associated factors, including SOX2, OCT4, HMGA2, KLF4, and BACH1 [30]. miR-142-3p also increased paclitaxel sensitivity by targeting CXCL12 and suppressing Wnt/β-catenin signaling, with corresponding reductions in P-glycoprotein and breast cancer resistance protein expression [37]. Collectively, miR-142-3p enhances chemosensitivity through multiple converging mechanisms, including regulation of autophagy, apoptosis, EMT, and drug efflux pathways as illustrated in Fig. 1.

**Table 1:** Effects of miR-142-3p on chemosensitivity across cancer types

Cancer type	Effect of miR-142-3p on chemosensitivity	Key target/mechanism	Reference
Non-small cell lung cancer (A549)	↑ Cisplatin sensitivity, ↑ Adriamycin sensitivity	↓ autophagy via HMGB1 inhibition	[64]
Breast cancer (MCF-7)	↑ Doxorubicin sensitivity	↓ autophagy via HMGB1 inhibition	[35]
Ovarian cancer (SKOV3)	↑ cisplatin sensitivity	↓ autophagy via SIRT1 inhibition	[31]
Lung cancer (H1299)	↑ Cisplatin sensitivity	↓ autophagy via SIRT1 inhibition	[49]
Breast cancer (MDA-MB-468)	↑ Docetaxel sensitivity	↓ EMT, ↓ stemness, ↑ apoptosis	[30]
Breast cancer (MCF-7)	↑paclitaxel sensitivity	↓ BCRP & P-gp via CXCL12/Wnt/β-catenin inhibition	[37]

## Extracellular vesicle-mediated functions of miR-142-3p

EVs are membrane-bound particles released by cells that facilitate intercellular communication by transferring proteins, lipids, and nucleic acids to recipient cells [71, 72]. Cancer cells often release more EVs than non-malignant cells, with further increases reported in drug-resistant cells and following exposure to therapeutic stress [73–77]. In cancer, EVs can promote disease progression by transferring tumor-supportive cargo between cells, removing tumor-suppressive molecules from cancer cells, and altering communication between cancer and stromal cells within the TME [19, 38, 78]. EV-mediated export of miR-142-3p may alter both its intracellular tumor-suppressive activity and its effects in recipient cells.

### Selective export of miR-142-3p in extracellular vesicles

Limited but emerging evidence suggests that miR-142-3p may be selectively enriched in EVs relative to donor cells in certain cancer contexts. Although this phenomenon has not been extensively investigated, several studies reported preferential enrichment of miR-142-3p in tumor-derived EVs. For example, miR-142-3p was enriched (> 4-fold compared to donor cells) in EVs derived from early lung adenocarcinoma (LAC) cells [39]. Similarly, RNA sequencing data from cervical cancer cell lines demonstrated higher levels of miR-142-3p in EVs compared to intracellular levels, consistent with selective EV-mediated export [44]. The mechanisms underlying selective miRNA incorporation into EVs are not fully understood but are thought to involve RNA-binding proteins that recognize specific sequence motifs. For instance, SYNCRIP was shown to bind miRNAs containing a GGCU motif and facilitate their selective loading into EVs. As miR-142-3p contains this motif, its enrichment in EVs is likely mediated, at least in part, through SYNCRIP-dependent sorting mechanisms [79].

This selective export of miR-142-3p has been proposed as a mechanism by which cancer cells reduce intracellular levels of miR-142-3p, thereby limiting its tumor-suppressive effects [4]. However, the extracellular presence of miR-142-3p within the TME may have additional functional consequences. EV-mediated delivery of miR-142-3p to recipient cells has been associated with tumor-supportive processes, including CAF activation, angiogenesis, and immune suppression collectively contributing to a pro-tumorigenic microenvironment [4, 38, 40, 42].

### Effects of EV-associated miR-142-3p within the tumor microenvironment

#### Cancer-associated fibroblast (CAF) activation

Fibroblasts represent a major and dynamic stromal population within the TME. Under pathological conditions, normal fibroblasts can undergo phenotypic reprogramming into cancer-associated fibroblasts (CAFs), which support tumor progression through extracellular-matrix remodeling, growth-factor secretion, invasion and metastasis, immune evasion, and treatment resistance [80].

Evidence linking EV-associated miR-142-3p to fibroblast activation remains limited. In a lung adenocarcinoma model, Lawson et al. (2019) demonstrated the transfer of miR-142-3p from cancer cells to fibroblasts through EVs [4]. This was evidenced by increased expression of canonical CAF markers, including α-smooth muscle actin (α-SMA) and platelet-derived growth factor receptor beta (PDGFRβ) [4]. The authors examined transforming growth factor-beta (TGF-β) signaling as a potential mechanism underlying this response because it is a well-established driver of fibroblast activation. However, neither treatment with miR-142-3p-enriched EVs nor direct overexpression of miR-142-3p in fibroblasts significantly altered TGF-β signaling [4]. The signaling pathways responsible for miR-142-3p-mediated fibroblast activation therefore remain unknown.

EV-mediated delivery of miR-142-3p also increased the migratory capacity of WI-38 fibroblasts. EVs derived from H1975 and H2073 lung cancer cells overexpressing miR-142-3p produced greater wound closure than control EVs [4]. Together with the increased expression of cancer-associated fibroblast markers, these findings suggest that EV-associated miR-142-3p may promote fibroblast activation and stromal remodeling. However, the evidence is currently limited to a single study conducted in one cancer context. Further studies are required to identify the underlying signaling pathways, determine whether this effect occurs across tumor types, and establish its relevance in vivo.

#### Angiogenesis

Angiogenesis, the formation of new blood vessels from pre-existing vasculature, is a hallmark of cancer that supports tumor growth and metastatic dissemination by supplying oxygen and nutrients [81]. This process is regulated by a balance between pro- and anti-angiogenic factors, which becomes disrupted in cancer to favor sustained vascular growth [82]. Key mediators such as vascular endothelial growth factor (VEGF), along with signaling pathways including VEGFR and TGF-β, regulate endothelial cell proliferation, migration, and survival within the TME [82]. Within the TME, EV-mediated transfer of microRNAs can influence intercellular communication and regulate angiogenic signaling [83].

Although the evidence remains limited, several studies support a pro-angiogenic role for EV-associated miR-142-3p [38, 84, 85]. Dickman et al. (2017) demonstrated that miR-142-3p-enriched EVs derived from oral cancer cells enhanced angiogenesis in HMEC-1 endothelial cells in vitro and in vivo [38]. Similarly, EVs derived from regulatory T cells promoted endothelial-cell proliferation, migration, cell-cycle progression, and tube formation in human umbilical vein endothelial cells [84]. Treatment with these EVs also increased miR-142-3p levels in recipient endothelial cells, supporting its transfer as EV cargo [84].

Mechanistically, miR-142-3p was shown to directly target the 3′-UTR region of TGFBR1, thereby reducing its expression and attenuating TGF-β/Smad signaling [38, 84]. This pathway normally promotes the expression of anti-angiogenic mediators, including thrombospondin-1 and soluble Fms-like tyrosine kinase-1 [86]. Thrombospondin-1 restricts endothelial-cell survival and pro-angiogenic signaling, whereas soluble Fms-like tyrosine kinase-1 acts as a decoy receptor that sequesters VEGF and limits VEGFR2 activation [87, 88]. Consequently, miR-142-3p-mediated suppression of TGFBR1 may reduce anti-angiogenic signaling and promote vascular growth.

A pro-angiogenic effect of miR-142-3p has also been reported in a non-cancer model of choroidal neovascularization, in which its inhibition reduced vascular growth and microglial recruitment, partly through regulation VEGFA production [85]. Although this finding supports the angiogenic potential of miR-142-3p, its relevance to cancer-associated angiogenesis was not investigated.

Collectively, these findings suggest that EV-mediated transfer of miR-142-3p from cancer and immune cells may promote angiogenic remodeling within the TME. However, evidence remains limited to a small number of experimental models, and further studies are required to establish the prevalence, context dependence, and in vivo significance of this mechanism across cancer types.

#### Immune modulation

Beyond its effect on stromal activation and angiogenesis, miR-142-3p can regulate immune responses in the TME, with consequences that appear to depend on the cellular source, recipient cell, and mode of transfer. However, evidence for EV-mediated immune regulation by miR-142-3p remains limited.

In antigen-presenting cells (APCs), miR-142-3p has been associated with immunosuppressive effects. Naqvi et al. reported that overexpression of miR-142-3p in macrophages and dendritic cells reduced antigen processing and impaired antigen uptake and clearance [42]. These effects were associated with increased expression of programmed death-ligand 1 (PD-L1), an immune-checkpoint protein that suppresses T-cell activation through binding to programmed cell death protein 1 (PD-1). The involvement of this pathway was supported by the restoration of T-cell proliferation following PD-L1 blockade. In addition, miR-142-3p suppressed Th1-associated cytokines, including TNF-α and IFN-γ. These findings indicate that miR-142-3p can impair antigen presentation and attenuate subsequent T-cell responses [42]. Although this study did not examine EV-mediated delivery or cancer-specific immunity, it identifies a potential mechanism through which tumor-derived EV-associated miR-142-3p could suppress APC and T cell function to evade immune detection. Furthermore, cancer-cell-derived miR-142-3p has been reported to be transferred to M1 macrophages in hepatitis B virus-associated hepatocellular carcinoma to promote ferroptosis thereby promoting tumor progression [89].

miR-142-3p has also been shown to enhance anti-tumor immune responses in a context-dependent manner. In glioblastoma, EVs derived from M1 macrophages were enriched in miR-142-3p and transferred it to tumor cells [55]. This delivery suppressed HMGB1 expression, reduced STAT3 activation, and downregulated PD-L1, thereby alleviating tumor-cell-mediated immune suppression [55]. Similarly, in hepatocellular carcinoma, intracellular miR-142-3p enhanced antitumor immunity by targeting ASH1L, although EV-mediated transfer was not investigated [90]. High ASH1L expression was associated with reduced expression of cytotoxic immune genes, including *GZMH*, *NKG7*, *CCL16*, and *CD8B*, as well as decreased immune cell infiltration. Suppression of ASH1L by miR-142-3p therefore promoted immune activity and recruitment.

Collectively, these findings highlight the context-dependent role of miR-142-3p in immune regulation. In APCs, miR-142-3p impaired antigen presentation and increased PD-L1 expression, whereas in tumor cells it suppressed immunoregulatory pathways involving HMGB1/STAT3 and ASH1L. However, the current evidence is derived from a small number of studies employing different cancer models and experimental approaches, several of which did not investigate EV-mediated transfer. A knowledge gap therefore remains regarding how the cellular source, EV-mediated delivery, recipient immune-cell type, and cancer context determine the immunological effects of miR-142-3p. Addressing these questions will be essential before its immune-modulatory or EV-mediated functions can be therapeutically targeted.

### Potential involvement of EV-associated miR-142-3p in cancer-associated cachexia

Although not traditionally considered a core component of the TME, systemic effects such as skeletal muscle loss have important consequences for cancer progression, treatment tolerance, and patient outcomes [91]. Cancer-associated cachexia is a complex condition characterized by unintended weight loss and progressive loss of skeletal muscle mass. It occurs in approximately 50–80% of patients with advanced-stage disease and is associated with reduced treatment tolerance and poorer overall survival [92–94]. Its pathophysiology involves metabolic dysregulation, increased energy expenditure, chronic inflammation, and tumor-derived factors such as proteolysis-inducing factor and lipid-mobilizing factor [95, 96]. Cachexia is also associated with systemic metabolic and immune dysregulation involving inflammatory mediators such as IL-6 [97–99].

EVs can contribute to cachexia through the transfer of tumor-derived cargo to skeletal muscle [100]. A direct role for tumor-derived EV miR-142-3p in cancer-associated cachexia has not yet been demonstrated. However, findings from non-cancer muscle-wasting models indicate that miR-142-3p can impair myogenic differentiation and promote muscle atrophy, providing a rationale for exploring this potential relationship.

Increased miR-142-3p expression has been reported in muscle-wasting conditions, including denervation-induced atrophy and muscular dystrophies, although these associations do not establish a functional role [101–103]. Mechanistic evidence from a non-cancer model indicates that miR-142-3p can impair myogenic differentiation. In C2C12 myoblasts, miR-142-3p targeted myocyte enhancer factor 2 A (MEF2A), a transcription factor that cooperates with MyoD to regulate muscle-specific gene expression [40]. Overexpression of miR-142-3p reduced cell proliferation, disrupted cell-cycle progression, and inhibited myotube formation, accompanied by reduced expression of MyoD, myogenin, and myosin heavy chain [40].

**Fig. 2:**
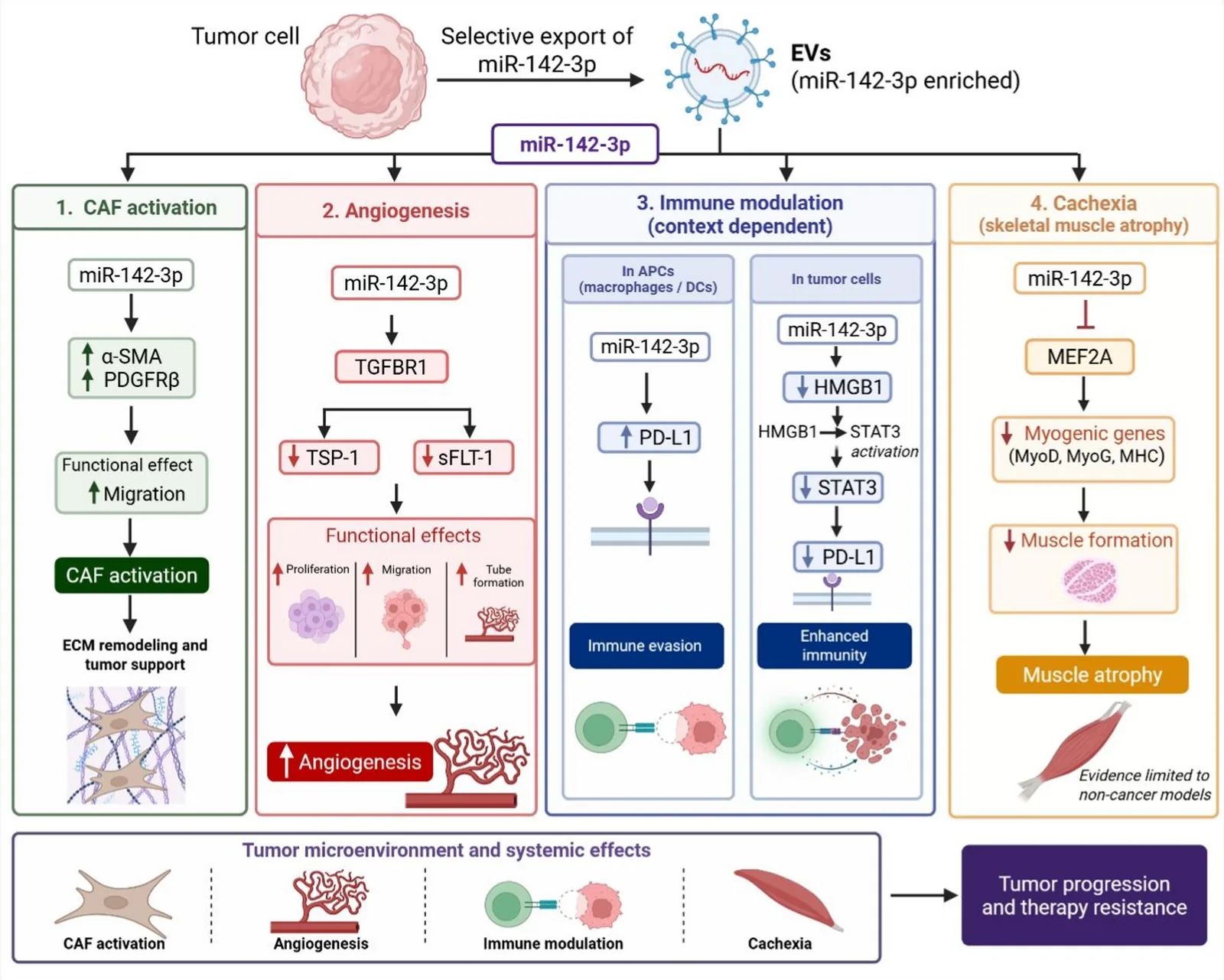
EV-mediated roles of miR-142-3p in the tumor microenvironment and systemic effects. Tumor cells export miR-142-3p via EVs, enabling its transfer to recipient cells. EV-associated miR-142-3p promotes cancer-associated fibroblast activation and angiogenesis, modulates immune responses in a context-dependent manner through PD-L1 regulation, and may contribute to muscle atrophy by impairing myogenesis. Collectively, these effects support tumor progression and therapy resistance. *Figure created with Biorender.com*

Consistent with these in vitro findings, knockdown of miR-142-3p alleviated muscle atrophy following nerve injury in vivo, increasing muscle mass, strength, and myofiber diameter [40]. These effects were accompanied by increased numbers of proliferating Pax7⁺ satellite cells, reduced expression of the atrophy-related genes *Atrogin-1*,* MuRF-1*, and *Nedd4*, and increased expression of myogenic markers. The protective effects of miR-142-3p knockdown were partially reversed by concurrent MEF2A knockdown, supporting the involvement of MEF2A-dependent myogenic pathways [40]. Collectively, these non-cancer findings raise the hypothesis that tumor-derived EV-associated miR-142-3p could contribute to skeletal muscle loss by suppressing myogenic differentiation and promoting atrophic signaling (Fig. 2).

## Therapeutic potential and future perspectives

miR-142-3p has context-dependent functions in cancer. Within cancer cells, it predominantly acts as a tumor suppressor by reducing proliferation, promoting apoptosis, suppressing EMT, and improving treatment response through pathways involving HMGB1, Wnt/β-catenin, and SIRT1-associated autophagy [3, 30–33]. However, its selective export through EVs may reduce its intracellular activity while enabling transfer to recipient cells. EV-associated miR-142-3p has been linked to CAF activation, angiogenesis, and immune regulation [4, 38, 42, 55]. Beyond the local tumor environment, miR-142-3p may also contribute to systemic effects, including the regulation of myogenic pathways linked to cachexia. However, current evidence remains limited and is largely derived from non-cancer models [40].

Given the intracellular tumor suppressive effects of miR-142-3p, restoring it within cancer cells could represent a therapeutic strategy. Synthetic miR-142-3p mimics may restore the regulation of oncogenic targets and improve treatment response. Preclinical studies have shown favourable results of miRNA mimics in several cancer types [104–106]. However, miRNA mimic therapies remain at an early stage of clinical development, and none has yet received regulatory approval [107, 108]. Current challenges associated with miRNA mimics include nuclease-mediated degradation, inefficient cellular uptake and endosomal escape, off-target effects, and inadequate tissue and cell-type specificity [109]. These barriers may be partly addressed through chemical modifications that improve miRNA stability and engineered nanocarrier-based delivery systems that protect the mimic and enhance cellular uptake. EVs, including exosomes, have emerged as promising biological nanocarriers because they can protect miRNA cargo and facilitate intercellular delivery [110–113]. Although further optimization and clinical validation are required, continued advances in EV engineering and miRNA loading provide a promising foundation for the development of miR-142-3p-based therapies.

An alternative therapeutic strategy may be to inhibit EV formation or release, potentially retaining miR-142-3p within cancer cells while reducing its transfer to recipient cells in the TME. Several compounds have been investigated as inhibitors of EV biogenesis and release by targeting various pathways involved in these processes, including nSMase2-dependent ceramide production [114–118]. nSMase2 generates ceramide from sphingomyelin, promoting intraluminal vesicle formation and exosome release. EV uptake by recipient cells could also be inhibited by targeting endocytic pathways [117, 119]. For example, inhibition of macropinocytosis using EIPA or targeting EGFR signaling with erlotinib or cetuximab has been shown to reduce EV uptake in oral squamous cell carcinoma [120].

Although these findings are promising, most EV-targeting strategies remain at the preclinical stage and require further evaluation in clinical studies. An important limitation is that currently available inhibitors are not specific to tumor-derived EVs and may disrupt physiologically important EV-mediated communication in healthy tissues. Developing approaches that selectively target tumor-associated EV pathways, or restrict inhibitor activity to the tumour site, will be important for clinical translation.

The context-dependent activity of miR-142-3p presents an additional translational challenge. Although restoring miR-142-3p within cancer cells may suppress tumor progression, unintended delivery to endothelial cells or fibroblasts could promote angiogenesis or CAF activation. Future strategies must therefore combine efficient miR-142-3p restoration with precise tumor- and cell-specific delivery. Encouragingly, continued advances in targeted nanocarriers and engineered EVs provide opportunities to achieve increased specificity. Together, these developments may enable the tumour-suppressive potential of intracellular miR-142-3p to be harnessed while limiting its unwanted effects in the TME.

## Conclusion

miR-142-3p holds considerable therapeutic potential as a context-dependent regulator of cancer progression and treatment response. Within cancer cells, it predominantly exerts tumor-suppressive effects; however, its EV-mediated export may reduce these intracellular effects while promoting CAF activation, angiogenesis, and immune modulation within the TME. EV-associated miR-142-3p may also contribute to systemic effects relevant to cancer cachexia, although this relationship remains insufficiently explored. Therapeutic strategies aimed at restoring miR-142-3p within cancer cells or modulating its EV-mediated transfer are therefore promising but will require precise cell-specific targeting. Further clarification of its intracellular, extracellular, and systemic functions, together with the development of cell-specific delivery and EV-targeting strategies, may ultimately enable its therapeutic potential to be safely and effectively harnessed.

## Data Availability

No datasets were generated or analysed during the current study.
